# Impact of Pathological Lymphovascular Invasion of Lung Adenocarcinoma after Induction Chemoradiotherapy

**DOI:** 10.5761/atcs.oa.25-00242

**Published:** 2026-07-01

**Authors:** Takafumi Kabuto, Toshi Menju, Shigeto Nishikawa, Kazuhiro Terada, Akihiko Yoshizawa, Hiroshi Date

**Affiliations:** 1 Department of Thoracic Surgery, Kyoto University Hospital, Kyoto, Kyoto, Japan; 2 Department of Thoracic Surgery, Japanese Red Cross Wakayama Medical Center, Wakayama, Wakayama, Japan; 3 Department of Diagnostic Pathology, Toyooka Public Hospital, Toyooka, Hyogo, Japan; 4 Department of Diagnostic Pathology, Kyoto University Hospital, Kyoto, Kyoto, Japan; 5 Department of Diagnostic Pathology, Nara Medical University, Kashihara, Nara, Japan; 6 Division of Thoracic Surgery, Duke University Medical Center, Durham, NC, USA

**Keywords:** locally advanced lung cancer, induction chemoradiotherapy, lymphovascular invasion

## Abstract

**Purpose:**

We investigated the clinical significance of lymphovascular invasion (LVI) in locally advanced lung adenocarcinoma after induction chemoradiotherapy (CRT).

**Methods:**

We retrospectively reviewed patients with completely resected lung adenocarcinoma after induction CRT from 2007 to 2021 at our institution. We evaluated the effects of LVI on recurrence-free survival (RFS) and overall survival (OS).

**Results:**

Fifty-six patients were included. The reasons for induction CRT were superior sulcus tumor (n = 11, 19.6%), direct invasion of other organs (n = 7, 12.5%), bulky N1 (n = 3, 5.4%), and N2 (n = 35, 62.5%). A pathological complete response was observed in 6 patients (10.7%). LVI was present in 14 patients (25.0%) and absent in 42 (75.0%). The LVI-positive group had significantly worse 5-year RFS (7.8% vs. 49.7%, p <0.001) and OS (35.2% vs. 73.5%, p = 0.003) than the LVI-negative group. Multivariate analysis revealed that LVI was a significant prognostic factor for both RFS (hazard ratio [HR], 3.91; 95% confidence interval [CI], 1.64–9.32, p = 0.002) and OS (HR, 5.75; 95% CI, 1.92–17.20, p = 0.002).

**Conclusions:**

LVI can be a poor prognostic factor and an important biomarker for assessing the effectiveness of multimodal treatment in lung adenocarcinoma.

## Introduction

Lung cancer remains one of the leading causes of cancer-related death worldwide.^1)^ Surgical resection alone is often insufficient for locally advanced lung cancers because of the high recurrence rate and limited operability. Therefore, perioperative multimodal therapy is considered necessary. Preoperative chemoradiotherapy (CRT) is widely used for locally advanced lung cancers to reduce the tumor burden and improve resectability.^2–5)^ The pathological residual rate is regarded as an important prognostic factor in patients with non-small-cell lung cancer receiving preoperative treatments, including induction CRT.^2)^ However, other pathological findings after neoadjuvant therapy have received little attention as potential biomarkers. Lymphovascular invasion (LVI) is a known adverse prognostic factor in early-stage lung cancers, but its clinical role in locally advanced cases, especially after induction therapy, has not been elucidated.^6–8)^ In the present study, we aimed to examine the prognostic significance of LVI in lung adenocarcinoma after induction CRT.

## Materials and Methods

### Patients

We retrospectively reviewed the data of patients with completely resected lung cancers after induction CRT from 2007 to 2021 at Kyoto University Hospital. Induction CRT was administered concurrently, followed by surgical resection. The clinical indication for neoadjuvant therapy was to achieve negative surgical margins and improve the rate of complete resection, and it was applied to a broad range of clinical conditions, including T3 or T4 tumors and bulky N1 or N2 disease.^4,5)^ Nodal staging was diagnosed according to radiographical (contrast-enhanced computed tomography or positron emission tomography) or histopathological (endobronchial ultrasound–transbronchial needle aspiration or mediastinoscopy) findings, as appropriate. Neoadjuvant tyrosine kinase and immuno-checkpoint inhibitor were not approved during this period.

We enrolled patients with lung adenocarcinomas diagnosed based on the pre- or postoperative pathological specimens. Patients who underwent salvage surgery after systemic chemotherapy or definitive CRT for initially unresectable lung cancer were excluded. Pathological diagnoses were revised according to the 5th World Health Organization classification of thoracic tumors and the 8th TNM classification by 2 pulmonary pathologists. LVI was defined as the presence of lymphatic or vascular invasion. We pathologically identified lymphatic and vascular invasion in postoperative specimens mainly on hematoxylin and eosin-stained slides. In addition, in some cases, lymphatic and vascular invasion were evaluated using immunohistochemical D2-40 staining and Elastica van Gieson staining, respectively.

The endpoints were recurrence-free survival (RFS) and overall survival (OS). RFS was defined as the period from surgery to the date of death or clinical diagnosis of primary tumor recurrence, and OS as the period from surgery to death. The data-cutoff date for this analysis was July 21, 2023. Patients lost to follow-up without any events were censored at the last known follow-up data. The residual rate of the primary tumor was also assessed by major pathological response (MPR) and pathological complete response (pCR). MPR and pCR were defined as a ≤10% proportion of surviving tumor cells and the absence of any active tumor cells remaining after neoadjuvant therapy, respectively. We evaluated the effects of the pathological findings on RFS and OS through multivariate analyses.

### Statistical analysis

The differences between groups with respect to the normally and non-normally distributed continuous variables were assessed using the t-test. Categorical variables were analyzed using the chi-squared test or Fisher’s exact test, as appropriate. The response rate of the primary tumor was illustrated with a waterfall plot. The Kaplan–Meier method and log-rank tests were used to analyze the RFS and OS curves. Cox proportional hazards analysis was performed by selecting the recurrence factors reported in previous studies, including ypStage, LVI, micropapillary pattern presence, solid pattern presence, MPR, and adjuvant chemotherapy. All p values were two-sided, and p <0.05 was set to denote statistical significance. Statistical analyses were performed using EZR, a graphical user interface for R (The R Foundation for Statistical Computing, Vienna, Austria).^9)^

## Results

Fifty-six patients with lung adenocarcinoma received induction CRT. The indications for induction CRT were superior sulcus tumor (n = 11, 19.6%), direct invasion of other organs (n = 7, 12.5%), bulky N1 (n = 3, 5.4%), and N2 (n = 35, 62.5%). LVI was present in 14 patients (25.0%, LVI-positive group) and absent in 42 patients (75.0%, LVI-negative group).

**Fig. 1:**
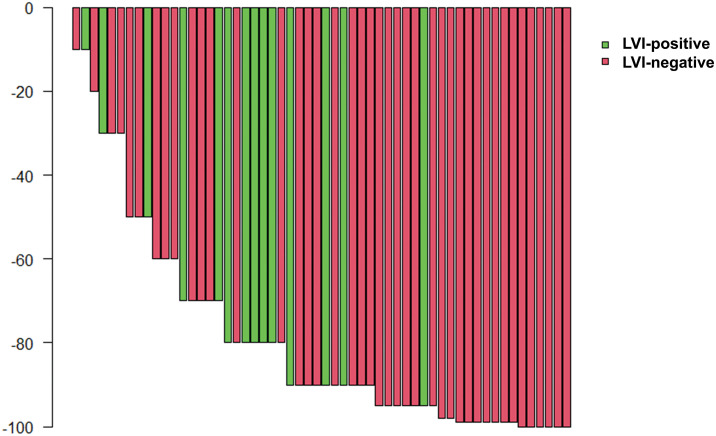
Waterfall plot of pathological response rate of the primary tumor. −100 indicates the pathological complete response. The green and red bars indicate the LVI-present and LVI-absent cases, respectively. LVI, lymphovascular invasion

The patients’ clinicopathological characteristics are summarized in **Table 1**. There were no significant differences between the 2 groups in age, sex, smoking history, induction chemotherapy regimen and reason, or radiation dose. **Figure 1** shows the response rate of the primary tumor. Six patients (10.7%) in the LVI-negative group achieved pCR. MPR was noted in 4 and 28 patients in the LVI-positive and LVI-negative groups, respectively. Although the pathological response rate was not statistically different (LVI-positive group: 71.1% vs. LVI-negative group: 81.6%, p = 0.167), the MPR rate was significantly higher in the LVI-negative group (28.6% vs. 66.7%, p = 0.027). The LVI-positive group was significantly more likely to have a solid pattern (57.1% vs. 23.8%, p = 0.044), whereas ypT, ypN, ypStage and the rate of micropapillary pattern were not significantly different between the groups. The adjuvant chemotherapy rate was also not significantly different (28.6% vs. 21.4%, p = 0.717).

**Table 1:** Patient clinicopathological characteristics

	All (n = 56)	LVI positive (n = 14)	LVI negative (n = 42)	p Value
Age, median (range)	64 (30–79)	60 (32–74)	64.5 (30–79)	0.381
Sex				
Male	36 (64.3%)	9 (64.3%)	27 (64.3%)	1
Smoking history				
Yes	34 (61.8%)	11 (78.6%)	23 (56.1%)	0.205
Follow up duration, median (range)	59.6 (0.9–147.6)	28.8 (4.7–72.2)	64.8 (0.9–147.6)	0.003
Induction regimen				
Cisplatin-based	26 (46.4%)	5 (35.7%)	21 (50.0%)	0.537
Carboplatin-based	30 (53.6%)	9 (64.3%)	21 (50.0%)	
Radiation dose (Gy), median (range)	50 (40–70)	50 (42–70)	50 (40–60)	0.334
Adjuvant chemotherapy				
Yes	13 (23.2%)	4 (28.6%)	9 (21.4%)	0.717
Induction reason				
Superior sulcus tumor	11 (19.6%)	1 (7.1%)	10 (23.8%)	0.228
Direct invasion	7 (12.5%)	2 (14.3%)	5 (11.9%)	
Bulky N1	3 (5.4%)	2 (14.3%)	1 (2.4%)	
N2	35 (62.5%)	9 (64.3%)	26 (61.9%)	
Type of resection				
Lobectomy	54 (96.4%)	13 (92.9%)	41 (97.6%)	0.441
Segmentectomy	1 (1.8%)	0 (0%)	1 (2.4%)	
Pneumonectomy	1 (1.8%)	1 (7.1%)	0 (0%)	
ypT				
0	6 (10.7%)	0 (0%)	6 (14.3%)	0.137
1	32 (57.1%)	7 (50.0%)	25 (59.5%)	
2	9 (16.1%)	5 (35.7%)	4 (9.5%)	
3	7 (12.5%)	2 (14.3%)	5 (11.9%)	
4	2 (3.6%)	0 (0%)	2 (4.8%)	
ypN				
0	29 (51.8%)	5 (35.7%)	24 (57.1%)	0.307
1	2 (3.6%)	1 (7.1%)	1 (2.4%)	
2	25 (44.6%)	8 (57.1%)	17 (40.5%)	
ypStage				
0	7 (12.5%)	0 (0%)	7 (16.7%)	0.278
1	11 (19.6%)	2 (14.3%)	9 (21.4%)	
2	10 (17.9%)	4 (28.6%)	6 (14.3%)	
3	28 (50.0%)	8 (57.1%)	20 (47.6%)	
Major pathological response				
Yes	32 (57.1%)	4 (28.6%)	28 (66.7%)	0.027
Pathological complete response				
Yes	6 (10.7%)	0 (0%)	6 (14.3%)	0.319
Micropapillary				
Presence	9 (16.1%)	4 (28.6%)	5 (11.9%)	0.206
Solid				
Presence	18 (32.1%)	8 (57.1%)	10 (23.8%)	0.044
Thirty-day mortality	1 (1.8%)	0 (0%)	1 (2.4%, interstitial pneumonia exacerbation)	1

LVI, lymphovascular invasion

**Fig. 2:**
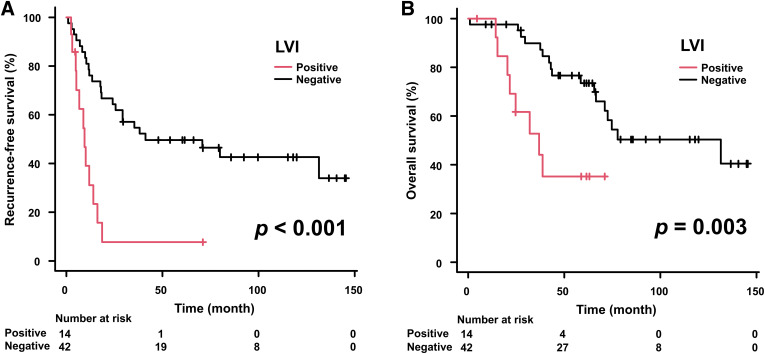
Prognoses from surgery. (**A**) Recurrence-free survival and (**B**) overall survival (red line: the LVI-positive group; black line: the LVI-negative group). LVI, lymphovascular invasion

**Fig. 3:**
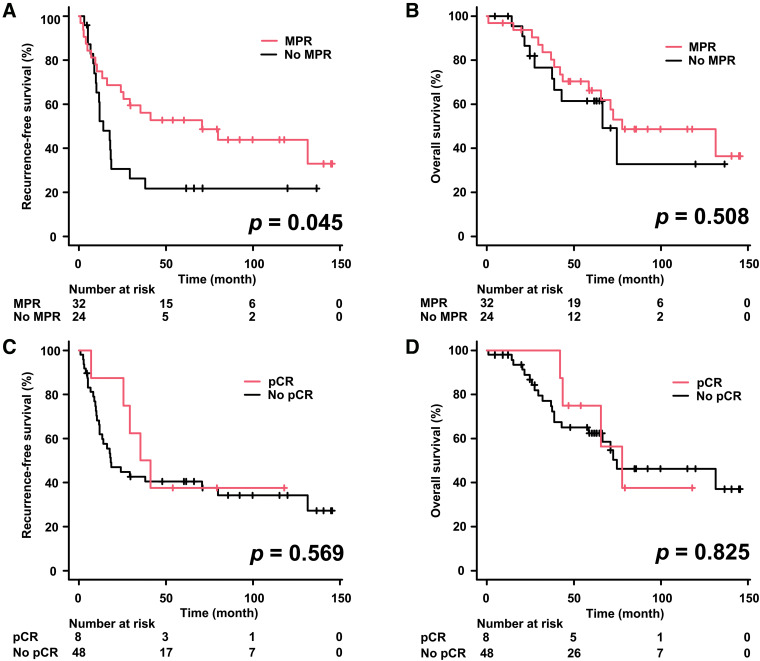
Prognoses from surgery by pathological response. (**A**) Recurrence-free survival and (**B**) overall survival of MPR. (**C**) Recurrence-free survival and (**D**) overall survival of pCR. MPR, major pathological response; pCR, pathological complete response

The 5-year RFS and OS for the entire cohort were 39.8% and 64.0%, respectively. The LVI-positive group had the significantly worse both of RFS (7.8% vs. 49.7%, p <0.001; median, 9.7 vs. 41.9 months) and OS (35.2% vs. 73.5%, p = 0.003; median, 37.6 vs. 133.3 months) compared with the LVI-negative group (**Fig. 2**). When stratified by the residual rate of the primary tumor, MPR was associated with a significant difference in RFS (5-year RFS: 52.8% vs. 21.8%; median, 71.8 vs. 14.3 months; p = 0.045) but not in OS (66.3% vs. 61.4%; median, 78.9 vs. 67.3 months; p = 0.508), while pCR was not associated with significant differences in either RFS (37.5% vs. 40.4%; median, 38.9 vs. 18.7 months, p = 0.569) or OS (75.0% vs. 62.4%; median, 78.9 vs. 75.8 months; p = 0.825) (**Fig. 3**).

The results of the Cox proportional hazards analysis are shown in **Table 2**. LVI was identified as an independent risk factor for both RFS (hazard ratio [HR], 3.91; 95% confidence interval [CI], 1.64–9.32; p = 0.002) and OS (HR, 5.75; 95% CI, 1.92–17.20; p = 0.002).

**Table 2:** Multivariate analysis

	Recurrence-free survival			Overall survival		
	Hazard ratio	95% Confidence interval	p Value	Hazard ratio	95% Confidence interval	p Value
ypStage	1.08	0.70–1.67	0.725	0.93	0.55–1.56	0.770
Lymphovascular invasion	3.91	1.64–9.32	0.002	5.75	1.92–17.20	0.002
Micropapillary pattern presence	1.01	0.38–2.74	0.979	0.27	0.06–1.35	0.111
Solid pattern presence	0.76	0.33–1.78	0.527	0.51	0.17–1.54	0.234
Major pathological response	0.81	0.30–2.21	0.679	0.67	0.21–2.17	0.505
Adjuvant chemotherapy	0.93	0.39–2.22	0.863	1.46	0.52–4.11	0.474

## Discussion

The present study demonstrated the significant clinical impact of LVI on the prognosis of patients with lung adenocarcinoma after preoperative CRT. Patients with resected lung adenocarcinoma who exhibited LVI after CRT had a markedly worse prognosis than those without LVI. Evidence regarding the prognostic value of LVI in locally advanced lung cancer is scarce, particularly in cohorts treated with CRT, and, therefore, our findings add clinically relevant information in this context.

LVI is a well-established adverse prognostic risk factor in early-stage lung cancers, as well as in other cancers such as colon and breast cancers.^6–8,10,11)^ The residual pathological findings after CRT are also considered informative; for example, in esophageal squamous cell carcinoma, which is commonly treated with neoadjuvant chemotherapy, the presence of LVI has been associated with unfavorable outcomes.^12)^ Our findings suggest that the presence of LVI in lung adenocarcinoma after CRT is similarly associated with a significantly worse prognosis, indicating that LVI may serve as a biomarker of aggressive tumor biology. Although previous studies suggest that the frequency of LVI in locally advanced lung cancer is relatively high,^7,8,13)^ the relatively lower frequency of LVI observed in our cohort may be attributable to treatment-related changes after CRT. Histopathological subtypes such as micropapillary or solid patterns are recognized as an adverse prognostic factors in early-stage lung adenocarcinoma.^14)^ However, this study showed that these subtypes were not independent risk factors in the case of locally advanced lung adenocarcinoma after CRT, and LVI may be a more reliable biomarker in treatment-exposed tumors.

The treatment strategy based on the pathological findings after the preoperative therapy has not been established. Although our data did not directly evaluate the adequacy of CRT or the benefit of adjuvant therapies, future studies should investigate whether additional systemic treatment could improve outcomes in this high-risk population with LVI, even as perioperative treatment standards continue to evolve. Perioperative immunotherapy is increasingly expected to play a central role in treatment strategies for locally advanced lung cancer.^15)^ Currently, pCR and MPR are the only well-established biomarkers, while data on other potential biomarkers are lacking.^15)^ Our findings may have relevance even in the era of perioperative immunotherapy and warrant further investigation.

In this study, neither MPR nor pCR was associated with survival. Previous reports have shown that the residual rate of primary tumors is evaluated during the preoperative therapy, and pCR has prognostic value mainly in squamous cell carcinoma.^2)^ However, adenocarcinoma tends to exhibit poorer clinical and pathological responses to CRT, likely due to lower radiosensitivity.^16)^ Because our cohort included only adenocarcinomas, treatment responses may differ from those reported in studies with mixed histologies. In this context, LVI emerged as a more reliable prognostic biomarker for both RFS and OS.

This study has several limitations due to its retrospective, single-center design and modest sample size. Nodal staging was based on a combination of radiographic and histopathological assessments, which may have introduced heterogeneity in staging accuracy, although this reflects real-world clinical practice. Preoperative LVI status could not be evaluated; therefore, the influence of induction CRT on LVI remains unclear, and LVI assessment may have been affected by treatment-related changes. In addition, all patients were treated prior to the availability of perioperative immunotherapy. However, historical cohorts remain essential for interpreting current therapeutic outcomes and for defining pathological markers applicable across treatment eras. Future prospective, multi-institutional analyses are warranted to validate the prognostic role of LVI and to determine its utility in guiding risk-adapted perioperative strategies.

## Conclusions

LVI can be a significant adverse prognostic factor in patients with lung adenocarcinoma after induction CRT and may serve as a useful biomarker of aggressive tumor biology. Although our study did not establish the efficacy of additional treatments, future investigations should clarify whether perioperative immunotherapy or other systemic strategies can improve outcomes in this high-risk population.

## Declarations

### Data availability

The data that support the findings of this study are available from the corresponding author upon reasonable request.
